# Pan-EGFR Inhibitor Dacomitinib Resensitizes Paclitaxel and Induces Apoptosis via Elevating Intracellular ROS Levels in Ovarian Cancer SKOV3-TR Cells

**DOI:** 10.3390/molecules29010274

**Published:** 2024-01-04

**Authors:** Ye Jin Lim, Hee Su Kim, Seunghee Bae, Kyeong A So, Tae Jin Kim, Jae Ho Lee

**Affiliations:** 1 Department of Cosmetics Engineering, Konkuk University, 120 Neungdong-ro, Seoul 05029, Republic of Korea; yejin513513@naver.com (Y.J.L.); kjhumy2@naver.com (H.S.K.); sbae@konkuk.ac.kr (S.B.); 2 Department of Obstetrics and Gynecology, Konkuk University School of Medicine, Seoul 05030, Republic of Korea; joyfulplace@hanmail.net (K.A.S.); kimonc111@naver.com (T.J.K.)

**Keywords:** dacomitinib, pan-EGFR inhibitor, ovarian cancer, paclitaxel-resistance, reactive oxygen species

## Abstract

Paclitaxel is still used as a standard first-line treatment for ovarian cancer. Although paclitaxel is effective for many types of cancer, the emergence of chemoresistant cells represents a major challenge in chemotherapy. Our study aimed to analyze the cellular mechanism of dacomitinib, a pan-epidermal growth factor receptor (EGFR) inhibitor, which resensitized paclitaxel and induced cell cytotoxicity in paclitaxel-resistant ovarian cancer SKOV3-TR cells. We investigated the significant reduction in cell viability cotreated with dacomitinib and paclitaxel by WST-1 assay and flow cytometry analysis. Dacomitinib inhibited EGFR family proteins, including EGFR and HER2, as well as its downstream signaling proteins, including AKT, STAT3, ERK, and p38. In addition, dacomitinib inhibited the phosphorylation of Bad, and combination treatment with paclitaxel effectively suppressed the expression of Mcl-1. A 2′-7′-dichlorodihydrofluorescein diacetate (DCFH-DA) assay revealed a substantial elevation in cellular reactive oxygen species (ROS) levels in SKOV3-TR cells cotreated with dacomitinib and paclitaxel, which subsequently mediated cell cytotoxicity. Additionally, we confirmed that dacomitinib inhibits chemoresistance in paclitaxel-resistant ovarian cancer HeyA8-MDR cells. Collectively, our research indicated that dacomitinib effectively resensitized paclitaxel in SKOV3-TR cells by inhibiting EGFR signaling and elevating intracellular ROS levels.

## 1. Introduction

Ovarian cancer is a gynecological cancer with a high mortality and recurrence rate [1,2,3]. Ovarian cancer is generally diagnosed at an advanced stage, primarily due to the absence of screening tests [3]. As of the present, the standard strategy for treating ovarian cancer involves cytoreductive surgery and chemotherapy employing regimens based on platinum or taxane [4,5]. The emergence of chemoresistant ovarian cancer has posed challenges to the use of medications such as cisplatin and paclitaxel in the therapeutic approach for ovarian cancer patients [6,7].

Paclitaxel is a natural compound in a class of taxane drugs isolated from the bark of the Pacific tree (*Taxus brevifolia* Nutt), approved by the Food and Drug Administration (FDA) in 1992 as a chemotherapy for ovarian cancer [8,9]. Paclitaxel targets microtubules and facilitates the stable assembly of microtubules from β-tubulin heterodimers, thereby inhibiting the depolymerization of the microtubule, which ultimately prevents cell division and eventually induces apoptosis [9,10]. Paclitaxel also limits tumor angiogenesis and induces the expression of genes and cytokines that inhibit cell growth and apoptosis [11]. This pharmaceutical capacity against cancer has demonstrated efficacy across a spectrum of malignancies, with particular prominence in the treatment of ovarian and breast carcinomas [12,13]. Despite the anticancer efficacy of paclitaxel against diverse malignancies, paclitaxel therapy may fail to treat ovarian cancer. One of these causes is the emergence of chemoresistant ovarian cancer cells, but to date, the mechanisms of paclitaxel resistance are very complex and have not yet been fully elucidated [9]. According to previous studies, 90% of deaths in patients with advanced ovarian cancer are due to their nonresponsiveness to drugs and mutations in various genes that cause chemoresistance in ovarian cancer cells treated with standard anticancer drugs such as paclitaxel and cisplatin [14,15].

One of the mechanisms causing chemoresistance is the overexpression of ATP-binding cassette (ABC) transporter proteins [16]. The most characteristic ABC transporter protein for chemoresistance is P-glycoprotein (P-gp/ABCC1/MDR1) [17]. It has been reported that overexpression of P-gp has shown poor clinical results in patients with chemoresistant cancer [18,19,20]. Increased expression of P-gp in cancer cells can induce selective chemoresistance due to its ability to transport a variety of substrates, including vinblastine, etoposide, cisplatin, and paclitaxel [21].

The epidermal growth factor receptor (EGFR) comprises four distinct receptors containing EGFR (ErbB1), HER2 (ErbB2), HER3 (ErbB3), and HER4 (ErbB4). These EGFR receptors are membrane-bound glycoproteins with an intracellular tyrosine kinase domain and an extracellular ligand-binding domain [22,23]. Activation of these receptor tyrosine kinases results in proliferation, survival, adhesion, migration, and invasion and plays an important role in cancer [5]. There are six autophosphorylation sites (Tyr 992, 1045, 1068, 1086, 1148, and 1173) in the carboxy-terminal tail of EGFR protein. The binding of extracellular ligands induces activation of the intracellular kinase domain at these autophosphorylation sites, triggering the EGFR signaling pathway [24,25]. These residues serve as docking sites for intracellular signaling factors, including phospholipase Cγ1, c-Cbl, Grb2, and Shc [26,27,28,29]. These factors can directly regulate the activation of various intracellular signaling cascades, including PI3K/AKT, STAT3, and ERK pathways, and are involved in differentiation, proliferation, survival, and transformation [30,31,32]. In addition, mutations and abnormal expression of EGFR have been observed in different cancer types, including glioblastomas, non-small cell lung cancer (NSCLC), breast cancer, and ovarian cancer [33,34,35,36]. Recently, a study has documented that the enhancement of chemosensitivity in ovarian cancer to cisplatin can be increased by EGFR blockade [37,38].

Reactive oxygen species (ROS) contribute to intracellular physiological functions such as abnormal cell growth, metastasis, and resistance to cell death in cancer while increasing oxidative stress and inducing cell death [39,40,41]. Since the excessive accumulation of intracellular ROS can inflict significant damage on cancer cells, suppressing cancer cells through ROS induction is one of the primary anticancer strategies [42,43]. Through an extensive understanding of the biological mechanism, several anticancer drugs such as cisplatin, doxorubicin, motexafin, and paclitaxel can promote ROS generation directly or indirectly in cancer cells [44,45,46,47]. Also, the antineoplastic agent paclitaxel that induces excessive intracellular ROS may affect the viability of surrounding cells through a bystander effect [48].

Dacomitinib is a pan-EGFR inhibitor developed by Pfizer Inc. for the treatment of solid tumors [49]. Unlike the first-generation EGFR tyrosine kinase inhibitor (EGFR TKI) (gefitinib and erlotinib), which is a reversible inhibitor selectively targeting EGFR, dacomitinib, the second-generation EGFR TKI, is an irreversible inhibitor that has activity on three ErbB family kinase members (EGFR/HER1, HER2, and HER4) [50]. In previous studies, dacomitinib has shown higher anticancer effects in terms of progression-free survival and response periods than gefitinib in the primary treatment of EGFR mutant-positive NSCLC patients [51,52] and has demonstrated clinical activity in recurrent/metastatic cranial squamous cell carcinoma (RM-SCCHN) [53]. Furthermore, dacomitinib is effective in treating bladder cancer that expresses multiple HER family target receptors [54]. Previous studies on ovarian cancer have shown that dacomitinib reduces growth, clonogenic potential, and anoikis resistance, and induces apoptosis [38]. A combination treatment of dacomitinib and cisplatin has been shown to inhibit viability and promote apoptosis in cisplatin-resistant epithelial ovarian cancer cells [38].

Nonetheless, there is no research examining the effect of dacomitinib on paclitaxel-resistant ovarian cancer cells. In this study, we demonstrated that dacomitinib exhibits anticancer efficacy by enhancing the resensitization of paclitaxel-resistant ovarian cancer cells for the first time. This effect occurred through the inhibition of EGFR and its downstream signaling pathway, increasing intracellular ROS levels.

## 2. Results

### 2.1. Dacomitinib Induced Cytotoxicity in Paclitaxel-Resistant Ovarian Cancer SKOV3-TR Cells

**Figure 1:**
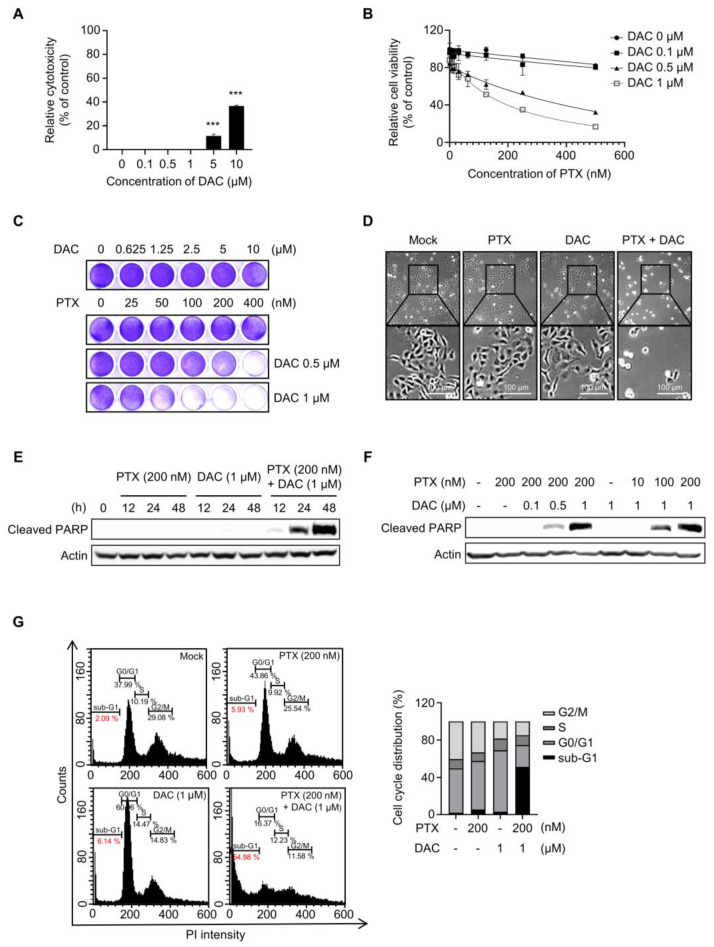
Cotreatment with dacomitinib and paclitaxel induced apoptosis in SKOV3-TR ovarian cancer cells. (**A**) SKOV3-TR cells were treated with various concentrations of dacomitinib (0, 0.1, 0.5, 1, 5, and 10 μM) for 48 h, and then LDH analysis was performed. (**B**) SKOV3-TR cells were cotreated with a combination of dacomitinib (0, 0.1, 0.5, and 1 µM) and serial dilutions of paclitaxel (0–500 nM) for 48 h. Cell viability was analyzed by WST-1 assay. (**C**) SKOV3-TR cells were cotreated with serial dilutions of paclitaxel (0–400 nM) and dacomitinib (0–10 μM) for 48 h, and then crystal violet assay was performed. (**D**) SKOV3-TR cells were subjected to treatments with DMSO (Mock), 200 nM paclitaxel, 1 µM dacomitinib, and a combination of 1 µM dacomitinib and 200 nM paclitaxel, each administered for 48 h. Subsequently, cellular observations were conducted under a microscope. (**E**) SKOV3-TR cells were subjected to treatments with 200 nM paclitaxel, 1 µM dacomitinib, and combination of 200 nM paclitaxel and 1 µM dacomitinib at various time intervals (0, 12, 24, and 48 h). Subsequently, the levels of cleaved PARP were analyzed by immunoblotting. (**F**) SKOV3-TR cells were treated with combination of dacomitinib (0, 0.1, 0.5, and 1 µM) and paclitaxel (0, 10, 100, and 200 nM) as indicated for 48 h, and then cleaved PARP was analyzed by immunoblotting. (**G**) SKOV3-TR cells were subjected to treatments with DMSO (Mock), 200 nM paclitaxel, 1 µM dacomitinib, and combination of 200 nM paclitaxel and 1 µM dacomitinib each for 48 h. Apoptotic cell death was analyzed by FACS analysis. Sub-G1 fraction (apoptotic cell fraction) was measured in percentages and shown as a graph. Actin was used as a loading control for each sample. The LDH assay data are presented as the mean percentage of control ± SD relative to the control. Values of *** *p* < 0.001 were considered statistically significant difference. DAC, dacomitinib; PTX, paclitaxel.

First, we evaluated the cytotoxic effect of dacomitinib (DAC) in SKOV3-TR cells. The cells were subjected to treatments with different concentrations of dacomitinib (0, 0.1, 0.5, 1, 5, and 10 µM) for 48 h, and then the cell cytotoxicity was examined by LDH assay. Figure 1A showed no significant cell cytotoxicity by dacomitinib at concentrations up to 1 μM, but treatment with 5 μM and 10 μM dacomitinib showed cytotoxicity of 1.57% and 36.75%, respectively. To determine the effect of dacomitinib on paclitaxel (PTX) resistance, cell viability was examined by WST-1 assay in SKOV3-TR cells at a concentration of 1 μM or less, which does not show cytotoxicity when dacomitinib is treated. SKOV3-TR cells were subjected to treatment with paclitaxel at serially diluted concentrations ranging from 0 to 500 nM, along with varying concentrations of dacomitinib (0, 0.1, 0.5, and 1 µM), for 48 h. The WST-1 assay showed that cell viability was significantly decreased in a dose-dependent manner in SKOV3-TR cells cotreated with dacomitinib and paclitaxel compared to paclitaxel alone (Figure 1B). Crystal violet assay also showed that cell viability decreased in a dose-dependent manner when these agents were treated (Figure 1C). Microscopic observations revealed that individual administrations of dacomitinib (1 μM) and paclitaxel (200 nM) did not change the morphology of SKOV3-TR cells. However, cotreatment with dacomitinib and paclitaxel led to a rounded and detached morphology of SKOV3-TR cells, indicating the induction of apoptosis (Figure 1D). Hence, we examined the levels of cleaved PARP, a widely recognized hallmark of apoptosis [55], following cotreatment with dacomitinib and paclitaxel in SKOV3-TR cells. Cleaved PARP was increased in SKOV3-TR cells cotreated with dacomitinib (1 μM) and paclitaxel (200 nM) in a time-dependent manner (Figure 1E). SKOV3-TR cells were treated with different concentrations of dacomitinib (0, 0.1, 0.5, and 1 μM) in combination with various concentrations of paclitaxel (0, 10, 100, and 200 nM). As a result, cleaved PARP was also induced by dacomitinib and paclitaxel in a dose-dependent manner, respectively (Figure 1F). Next, we confirmed the cytotoxic effect of the combination treatment of dacomitinib and paclitaxel in SKOV3-TR cells using FACS analysis. SKOV3-TR cells were treated separately or together with dacomitinib and paclitaxel for 48h. Then, cells were stained with propidium iodide staining and the sub-G1 fraction was measured. Figure 1G showed that the combination of paclitaxel and dacomitinib resulted in a higher sub-G1 fraction compared to the other sub-G1 fractions (2.09% in control cells, 5.93% in paclitaxel-treated cells, 6.14% in dacomitinib-treated cells, and 54.98% in the combination of dacomitinib and paclitaxel-treated cells). Collectively, these results indicate that dacomitinib restores paclitaxel sensitivity in paclitaxel-resistant ovarian cancer SKOV3-TR cells, leading to paclitaxel-induced cytotoxicity.

### 2.2. Dacomitinib Suppressed EGFR Signaling in Ovarian Cancer SKOV3-TR Cells

**Figure 2:**
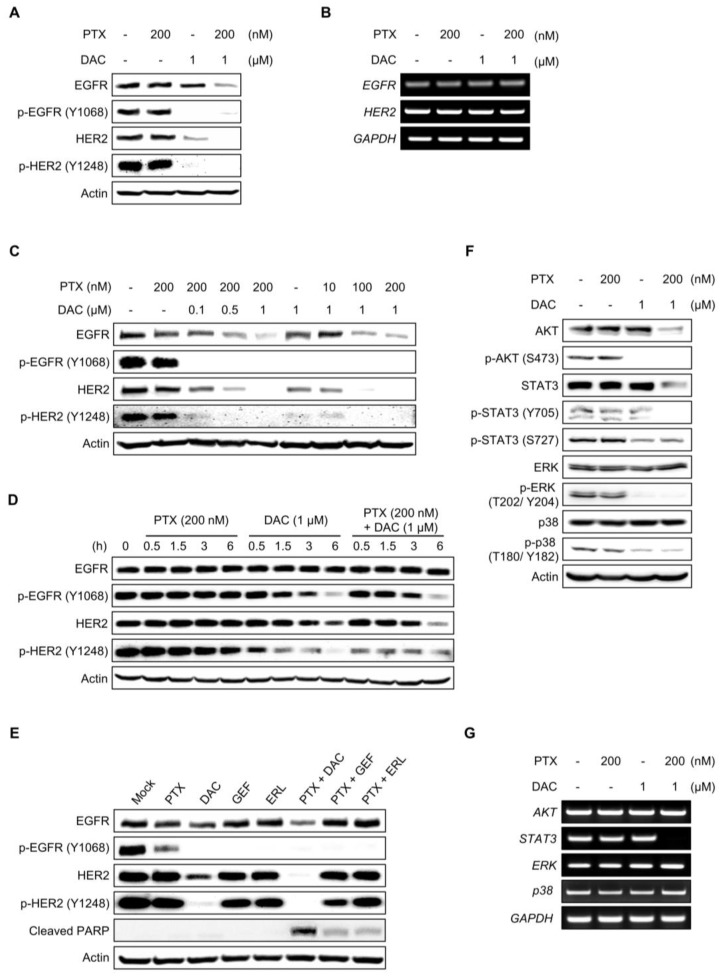
Dacomitinib suppressed EGFR and its downstream signaling in SKOV3-TR cells. (**A**) SKOV3-TR cells were subjected to treatments with 200 nM paclitaxel, 1 µM dacomitinib, and combination of 200 nM paclitaxel and 1 µM dacomitinib for 48 h. The expression of EGFR and HER2 and their phosphorylated forms (phosphorylated EGFR at Tyr1068 and phosphorylated HER2 at Tyr1248) were analyzed by immunoblotting. (**B**) Transcriptions of *EGFR* and *HER2* were examined by RT-PCR. (**C**) SKOV3-TR cells were cotreated with dacomitinib (0, 0.1, 0.5, and 1 µM) and paclitaxel (0, 10, 100, and 200 nM) as indicated for 48 h. The expression levels of EGFR and HER2 and their phosphorylation (phosphorylated EGFR at Tyr1068 and phosphorylated HER2 at Tyr1248) were examined by immunoblotting. (**D**) SKOV3-TR cells were subjected to treatments with 200 nM paclitaxel, 1 µM dacomitinib, and combination of 200 nM paclitaxel and 1 µM dacomitinib, and then the expression levels of EGFR and HER2 and their phosphorylation status (phosphorylated EGFR at Tyr1068 and phosphorylated HER2 at Tyr1248) were examined at different time points (0, 0.5, 1.5, 3, and 6 h). (**E**) SKOV3-TR cells were treated with 200 nM paclitaxel, 1 µM dacomitinib, 1 µM geftinib, and 1 µM erlutinib in the presence or absence of 200 nM paclitaxel as indicated for 48 h and then the expression of EGFR and HER2 and their phosphorylation (phosphorylated EGFR at Tyr1068 and phosphorylated HER2 at Tyr1248) were examined by immunoblotting. (**F**) SKOV3-TR cells were subjected to treatments with 200 nM paclitaxel, 1 µM dacomitinib, and combination of 200 nM paclitaxel and 1 µM dacomitinib for 48 h. Then, the expression of AKT, STAT, ERK, and p38 and their phosphorylation levels (phosphorylated AKT at Ser473, phosphorylated STAT3 at Tyr705, phosphorylated STAT3 at Ser727, phosphorylated ERK at Thr202/Tyr204, and phosphorylated p38 at Thr180/Tyr182) were analyzed by immunoblotting. (**G**) Transcriptions of *AKT*, *STAT3*, *ERK*, and *p38* were examined by RT-PCR. *GAPDH* was used as a loading control for the mRNAs in each sample. Actin was used as a loading control for each sample. PTX, paclitaxel; DAC, dacomitinib; GEF, gefitinib; ERL, erlotinib.

Dacomitinib is known to be a pan-EGFR inhibitor that targets not only EGFR but also HER2 and HER4 [56,57,58]. We investigated the effect of dacomitinib on EGFR and its downstream signaling in SKOV3-TR cells. Dacomitinib effectively inhibited the phosphorylation of EGFR at the Tyr1068 residue as well as the phosphorylation of HER2 at the Tyr1248 residue in SKOV3-TR cells (Figure 2A). However, dacomitinib did not affect the total mRNA levels of *EGFR* and *HER2* (Figure 2B). Previous studies have reported that paclitaxel can inhibit EGFR signaling by promoting the EGFR internalization/degradation mechanism [59,60]. Therefore, we investigated whether cotreatment with paclitaxel and dacomitinib could have a synergistic effect on the downregulation of EGFR signaling by promoting the suppression of receptors along with EGFR phosphorylation in SKOV3-TR cells. In Figure 2C, dacomitinib inhibited the phosphorylation of EGFR even at 0.1 µM treatment with paclitaxel in SKOV3-TR cells. Interestingly, dacomitinib did not show degradation of EGFR due to receptor internalization/degradation mechanism when treated alone at 1 µM (Figure 2C, lane 6), but receptor degradation occurred when treated with paclitaxel in a dose-dependent manner (Figure 2C, lanes 3–5). Moreover, dose-dependent treatment of paclitaxel induced EGFR degradation when treated with 1 µM dacomitinib (Figure 2C, lanes 7–9). The phosphorylation and expression of HER2 were also inhibited in SKOV3-TR cells by treatment with dacomitinib and paclitaxel (Figure 2C). The treatment with dacomitinib inhibited the phosphorylation of EGFR within 3 h, while phosphorylation of HER2 inhibition occurred earlier, within 1 h (Figure 2D). Next, we compared the EGFR inhibition and degradation activities of dacomitinib and the selective EGFR inhibitors gefitinib (GEF) and erlotinib (ERL) in SKOV3-TR cells [61,62]. Dacomitinib effectively inhibited the phosphorylation of both EGFR and HER2, whereas gefitinib and erlotinib only suppressed EGFR phosphorylation (Figure 2E). Interestingly, the combination treatment of dacomitinib and paclitaxel resulted in higher levels of cleaved PARP and enhanced EGFR degradation compared to the gefitinib/paclitaxel or erlotinib/paclitaxel combination treatments (Figure 2E). Subsequently, we investigated the effect of EGFR and its downstream signaling pathways upon treatment with dacomitinib and paclitaxel in SKOV3-TR cells. Immunoblotting analysis showed that dacomitinib effectively suppressed the phosphorylation of AKT, STAT3, ERK, and p38 MAPK (Figure 2F), which are EGFR downstream signaling factors that are well known to affect intracellular proliferation and survival pathways [63,64,65,66,67,68,69]. The transcriptional expressions of EGFR downstream factors (AKT, STAT3, ERK, and p38) were examined by RT-PCR in dacomitinib- and paclitaxel-treated SKOV3-TR cells. The results showed that dacomitinib did not affect the transcriptional expression of EGFR downstream factors (Figure 2G). Interestingly, only the transcription of STAT3 was inhibited in the combined treatment of dacomitinib and paclitaxel (Figure 2G). This suggests that the transcription of STAT3 might be inhibited at the transcriptional level during paclitaxel-mediated apoptotic cell death. These results suggest that dacomitinib can inhibit EGFR and its downstream signaling in SKOV3-TR cells, thereby inhibiting the expression and activation of factors associated with cellular proliferation, differentiation, and survival pathways.

### 2.3. Inhibition of HER2 Did Not Resensitize Paclitaxel in SKOV3-TR Cells

**Figure 3:**
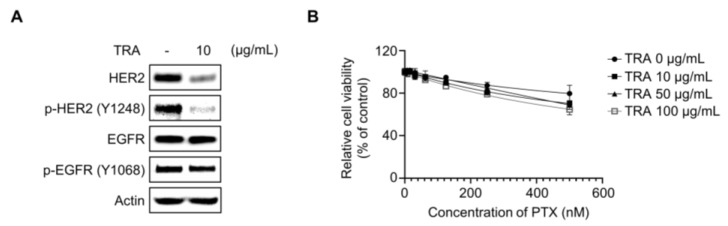
HER2 signaling did not affect the paclitaxel resensitization mechanism of dacomitinib in SKOV3-TR cells. (**A**) SKOV3-TR cells were treated with trastuzumab (10 µg/mL) for 48 h, and then the expression of EGFR and HER2 and their phosphorylation (phosphorylated EGFR at Tyr1068 and phosphorylated HER2 at Tyr1248) were examined by immunoblotting. (**B**) SKOV3-TR cells were cotreated with combination of a serial dilution of paclitaxel (0–500 nM) and trastuzumab (0, 10, 50, and 100 µg/mL) as indicated for 48 h. Cell viability analysis was performed by WST-1 assay. Actin was used as a loading control for each sample. TRA, trastuzumab.

The inhibition of EGFR and its downstream signaling by treatment of pan-EGFR inhibitor dacomitinib resulted in a reduction in cell viability in paclitaxel-treated SKOV3-TR cells (Figure 1 and Figure 2), leading us to further investigate whether selective HER2 inhibition affects paclitaxel resensitization in SKOV3-TR cells. We examined whether the selective HER2 inhibitor trastuzumab is able to resensitize paclitaxel in SKOV3-TR. Immunoblotting showed that treatment with 10 μg/mL trastuzumab (TRA) inhibited the phosphorylation of HER2 in SKOV3-TR cells while it did not downregulate the phosphorylation of EGFR (Figure 3A). As shown in Figure 3B, the selective inhibition of HER2 by trastuzumab did not inhibit cell viability in paclitaxel-treated SKOV3-TR cells, indicating that HER2 signaling does not affect paclitaxel resistance.

### 2.4. Dacomitinib Did Not Affect the Expression and Function of P-gp in SKOV3-TR Cells

**Figure 4:**
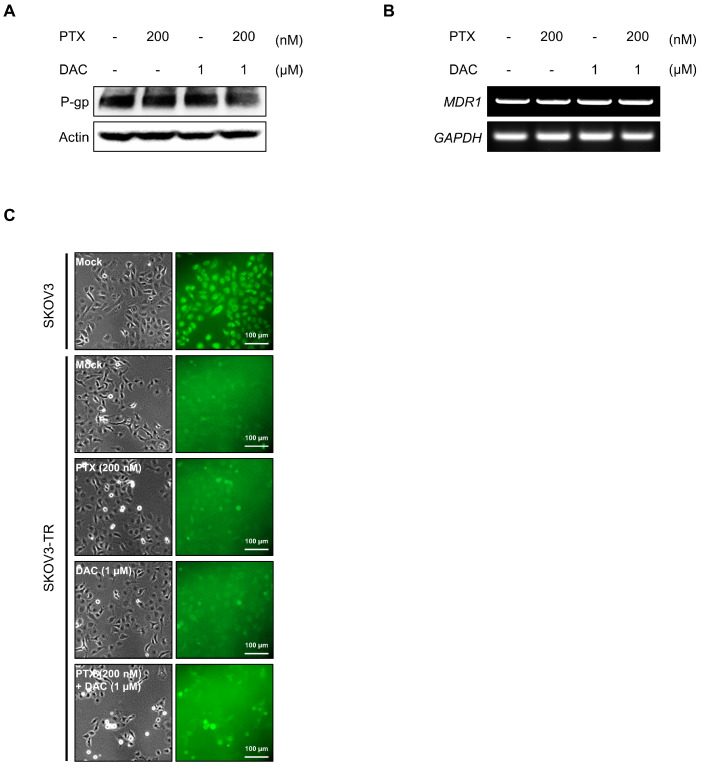
Dacomitinib did not affect the expression and function of P-gp in SKOV3-TR cells. (**A**) SKOV3-TR cells were subjected to treatments with 200 nM paclitaxel, 1 µM dacomitinib, and combination of 200 nM paclitaxel and 1 µM dacomitinib for 48 h. Then, the expression levels of P-gp were examined by immunoblotting. (**B**) Transcription levels of MDR1 were analyzed by RT-PCR in SKOV3-TR cells treated with paclitaxel and dacomitinib as indicated. (**C**) SKOV3-TR cells (7 × 10^5^) were seeded and further incubated for 24 h. Then, cells were subjected to treatments with DMSO (Mock), 200 nM paclitaxel, 1 µM dacomitinib, and a combination of 200 nM paclitaxel and 1 µM dacomitinib for 24 h. Fluo-3/AM solution was added and further reacted at 37 °C for 1 h, and then the fluorescences were visualized using an inverted fluorescence microscope. SKOV3 cells, the parent cells of SKOV3-TR, were used as paclitaxel-sensitive controls. Actin was used as a loading control for each sample. *GAPDH* was used as a loading control for the mRNAs in each sample. PTX, paclitaxel; DAC, dacomitinib.

We sought to determine how dacomitinib resensitizes paclitaxel in SKOV3-TR cells and induces paclitaxel-induced cell cytotoxicity. P-gp is a member of the ATP-dependent efflux transporters, which efflux a wide range of chemical anticancer drugs such as paclitaxel, cisplatin, and doxorubicin [70,71,72,73]. According to previous findings, the acquired overexpression of P-gp contributes to chemoresistance against anticancer drugs in various cancer types, including breast and ovarian cancers [17]. We tested whether dacomitinib affected the expression or function of P-gp in SKOV3-TR cells. The expression of P-gp was higher in SKOV3-TR cells than in the parent SKOV3 cells (Figure S1). Immunoblotting showed that treatment with dacomitinib alone or in combination with dacomitinib and paclitaxel did not affect the expression of P-gp in SKOV3-TR cells (Figure 4A,B). Next, we examined whether dacomitinib could affect the function of P-gp using the cell-permeant dye fluo-3/AM, a fluorescent substrate for P-gp [74,75]. SKOV3-TR cells were subjected to treatments with dacomitinib and paclitaxel alone or cotreated with dacomitinib and paclitaxel for 24 h, and then cells were additionally treated with 4 μM fluo-3/AM for 1 h. In Figure 4C, UV-microscopic observation showed that cell fluorescence was lower in SKOV3-TR cells than in SKOV3 cells due to the overexpression of P-gp. However, the cell fluorescence by fluo-3/AM did not increase with dacomitinib alone or in the combination treatment with dacomitinib and paclitaxel, indicating that dacomitinib does not affect the function of P-gp in SKOV3-TR cells (Figure 4C).

### 2.5. Dacomitinib Downregulated the Expression of Mcl-1 and the Phosphorylation of Bad in Paclitaxel-Treated SKOV3-TR Cells

**Figure 5:**
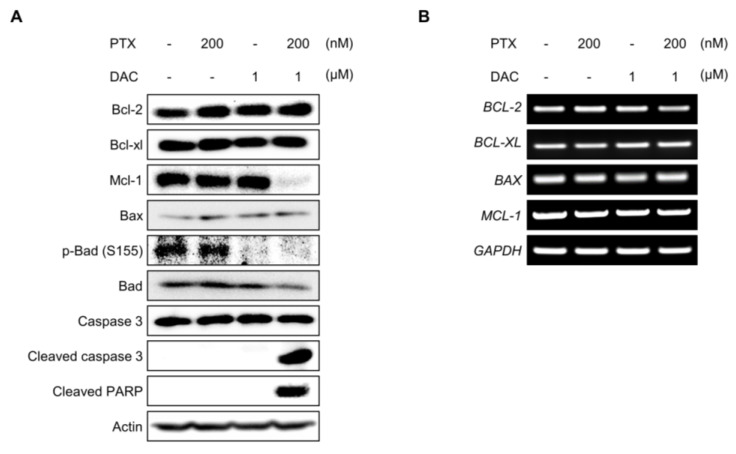
Dacomitinib suppressed the expression of Mcl-1 and the phosphorylation of Bad in paclitaxel-treated SKOV3-TR cells. (**A**) SKOV3-TR cells were subjected to treatments with 200 nM paclitaxel, 1 µM dacomitinib, and combination of 200 nM paclitaxel and 1 µM dacomitinib for 48 h. The expression levels of prosurvival Bcl-2 family proteins (Bcl-2, Bcl-xl, and Mcl-1), apoptosis effector Bcl-2 family protein (Bax), proapoptotic Bcl-2 family protein (Bad), and its phosphorylation (phospho-Bad at Ser155 residue) were examined by immunoblotting. The apoptotic mediators (caspase-3, cleaved caspase-3, and cleaved PARP) were also analyzed by immunoblotting. (**B**) Transcriptions of *BCL-2*, *BCL-XL*, *BAX*, and *MCL-1* were analyzed by RT-PCR. Actin was used as a loading control for each sample. *GAPDH* was used as a loading control for the mRNAs in each sample. PTX, paclitaxel; DAC, dacomitinib.

It has been reported that paclitaxel-induced apoptosis is associated with Bcl-2 family proteins [76,77,78]. The Bcl-2 family can regulate the apoptotic pathway in response to cellular stresses, and abnormalities of these proteins act as a barrier to cancer therapy [79,80,81]. We examined whether the expression and phosphorylation of Bcl-2 family proteins are affected by the apoptotic process induced by the combination treatment of dacomitinib and paclitaxel in SKOV3-TR cells. Interestingly, the cellular expression of Mcl-1 was drastically reduced upon the cotreatment of dacomitinib and paclitaxel, and the expression of cleaved caspase-3, the active form of caspase 3, and cleaved PARP were clearly increased (Figure 5A). Moreover, dacomitinib reduced the phosphorylation of Bad at Ser155 residue (Figure 5A). RT-PCR analysis revealed that the combination of dacomitinib and paclitaxel did not affect the transcriptional expression of *MCL-1* (Figure 5B), indicating that the expression of Mcl-1 was reduced by post-transcriptional regulation. These results suggested that the combination treatment of dacomitinib and paclitaxel may inhibit the expression of Mcl-1 and the phosphorylation of Bad through inhibition of EGFR and its downstream pathways, which increased the susceptibility of paclitaxel-induced apoptosis in SKOV3-TR cells.

### 2.6. Dacomitinib Increased Intracellular ROS Levels in Paclitaxel-Treated SKOV3-TR Cells

**Figure 6:**
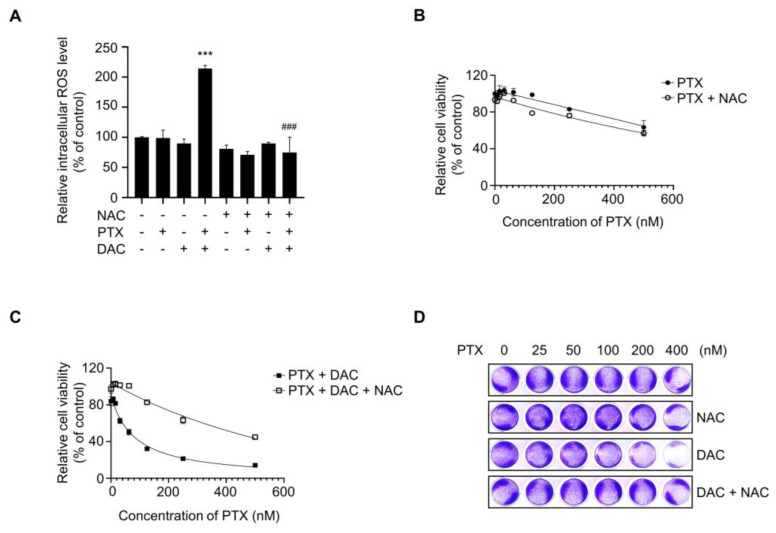
Dacomitinib increased intracellular ROS levels in paclitaxel-treated SKOV3-TR cells. (**A**) SKOV3-TR cells were subjected to treatments with 200 nM paclitaxel, 1 µM dacomitinib, and combination of 200 nM paclitaxel and 1 µM dacomitinib, with or without 5 mM N-acetylcysteine for 48 h. Cells were additionally treated with 10 µM DCFH-DA and further incubated for 30 min at 37 °C. Intracellular ROS levels were analyzed by measuring fluorescence values at excitation wavelengths of 485 nm and emission wavelengths of 520 nm using a fluorescence microplate reader. Relative intracellular ROS levels were calculated as the percentage of fluorescence values in the treatment group compared to those in the control group. (**B**) SKOV3-TR cells were subjected to treatments with a serial dilution of paclitaxel (0–500 nM) with or without 5 mM N-acetylcysteine for 48 h, and then cell viability was examined by WST-1 assay. (**C**) SKOV3-TR cells were subjected to treatments with combination of serial dilutions of paclitaxel (0–500 nM) and 1 µM dacomitinib with or without 5 mM N-acetylcysteine for 48 h, and then cell viability was examined by WST-1 assay. (**D**) SKOV3-TR cells were subjected to treatments with combination of serial dilutions of paclitaxel (0–400 nM), 1 μM dacomitinib, and 5 mM N-acetylcysteine as indicated for 48 h and then stained with 0.2% crystal violet solution for 1 h at room temperature. The DCFH-DA assay data are presented as the mean percentage of control ± SD relative to the control. Values of ***, ### *p* < 0.001 were considered statistically significant. PTX, paclitaxel; DAC, dacomitinib; NAC, N-acetylcysteine.

Several reports have indicated that induction of excessive intracellular ROS is one of the major mechanisms of paclitaxel-mediated apoptosis [48,82]. So, we investigated whether ROS were excessively generated during apoptosis induced by combination treatment of dacomitinib and paclitaxel in SKOV3-TR cells. In Figure 6A, DCFH-DA assay showed that the level of ROS in SKOV3-TR cells cotreated with dacomitinib and paclitaxel was significantly increased by more than 2.1-fold compared to the control cells. Also, the increased ROS by cotreatment with dacomitinib and paclitaxel was effectively reduced by additional treatment with N-acetylcysteine (NAC), a ROS scavenging agent (Figure 6A). Next, we used the WST-1 assay to test whether ROS scavenging with NAC in SKOV3-TR cells could affect cytotoxicity induced by combination treatment with dacomitinib and paclitaxel. The WST-1 assay showed that NAC had no effect on SKOV3-TR cells when treated with paclitaxel alone (Figure 6B), but it efficiently attenuated cell cytotoxicity in SKOV3-TR cells cotreated with dacomitinib and paclitaxel (Figure 6C). Additionally, crystal violet assay confirmed that ROS scavenging by NAC treatment inhibited cell death induced by cotreatment with dacomitinib and paclitaxel, thereby increasing cell viability in SKOV3-TR cells (Figure 6D). Collectively, our data indicated that the combination of dacomitinib and paclitaxel successfully induced cell cytotoxicity in SKOV3-TR cells through the excessive intracellular induction of ROS.

### 2.7. Dacomitinib Induced Cytotoxicity in Paclitaxel-Resistant Ovarian Cancer HeyA8-MDR Cells

**Figure 7:**
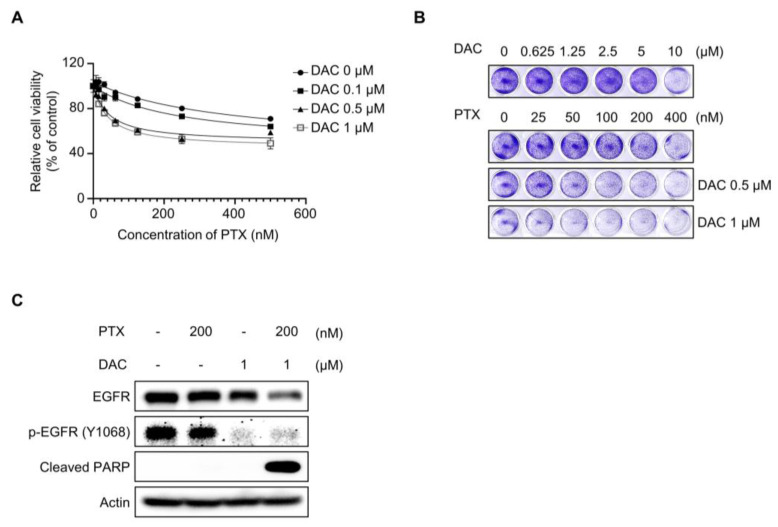
Dacomitinib resensitized paclitaxel in HeyA8-MDR ovarian cancer cells. (**A**) HeyA8-MDR cells were cotreated with a serial dilution of paclitaxel (0–500 nM) and specific concentrations of dacomitinib (0, 0.1, 0.5, and 1 µM) for 48 h. Cell viability was analyzed through WST-1 assay. (**B**) HeyA8-MDR cells were cotreated with serial dilutions of paclitaxel (0–400 nM) and dacomitinib (0–10 μM) for 48 h and then stained with 0.2% crystal violet solution for 1 h at room temperature. (**C**) HeyA8-MDR cells were subjected to treatments with paclitaxel (200 nM), dacomitinib (1 µM), and combination of paclitaxel (200 nM) and dacomitinib (1 µM) for 48 h. Immunoblotting was used to validate the expression levels of EGFR and its phosphorylation status (phosphorylated EGFR at Y1068). Actin was used as a loading control for each sample. PTX, paclitaxel; DAC, dacomitinib.

To investigate whether the restoration of paclitaxel sensitivity by dacomitinib is specific to the SKOV3-TR cell type, we examined cell viability assay in paclitaxel-resistant ovarian cancer HeyA8-MDR cells treated with dacomitinib and paclitaxel. In Figure 7A, the WST-1 assay showed that dacomitinib increased cell death in paclitaxel-treated HeyA8-MDR cells in a dose-dependent manner. Also, crystal violet assay confirmed that dacomitinib inhibited cell viability in paclitaxel-treated HeyA8-MDR cells (Figure 7B). The phosphorylation of EGFR at Tyr1068 residue was inhibited by treatment with dacomitinib, and apoptotic cell death was induced by a combination treatment of dacomitinib and paclitaxel (Figure 7C). These results indicated that the restoration of paclitaxel sensitivity in paclitaxel-resistant ovarian cancer cells is not a cell-type-dependent phenomenon.

## 3. Discussion

To date, the standard treatment of ovarian cancer is surgery and chemotherapy (platinum- and taxane-based chemical anticancer drugs) [83,84]. As highlighted by numerous studies, chemoresistance represents a significant obstacle in cancer therapy [1,6,15,83,85,86,87,88]. Overcoming chemoresistance is a crucial challenge within the field of cancer treatment because chemoresistance is associated with disease recurrence, metastasis, and impaired clinical outcomes in cancer patients [50,89,90,91].

Dacomitinib is a second-generation pan-EGFR inhibitor targeting EGFR, HER2, and HER4 receptor tyrosine kinases that has been approved for the first-line therapeutics of metastatic NSCLC with EGFR mutation [49]. Dacomitinib irreversibly inhibits the kinase activity of EGFR through covalent binding to Cys797 residue at the ATP-binding site of EGFR in the intracellular kinase domain [92,93]. It has been reported that dacomitinib has effective anticancer activity in various cancers, including EGFR-mutation-positive NSCLC, HER2-positive gastric cancer, HER2-amplified breast cancer, human bladder cancer, and cetuximab-resistant head and neck cancer cell lines [51,54,57,94,95]. However, research on dacomitinib in chemoresistance is insufficient compared to its own anticancer effects. In this study, we demonstrated the effect of dacomitinib on paclitaxel resensitization and the mechanism of action in paclitaxel-resistant ovarian cancer cells (Figure 1). The restoration of paclitaxel sensitivity by dacomitinib in paclitaxel-resistant ovarian cancer HeyA8-MDR cells (Figure 7) suggests the potential of dacomitinib as an effective agent for suppressing chemoresistance in ovarian cancer via inhibiting the antioxidant signaling pathway.

We showed that treatment with dacomitinib at a sublethal concentration of 1 μM effectively inhibited the phosphorylation of EGFR and its downstream signaling proteins in SKOV3-TR cells in the presence or absence of paclitaxel (Figure 2A,F). Therefore, we concluded that inhibition of EGFR phosphorylation itself did not induce apoptosis, but the decrease in cell survival signaling induced apoptotic cell death when cotreated with paclitaxel. Our data showed that dacomitinib simultaneously inhibited not only phosphorylation of EGFR but also phosphorylation of HER2 (Figure 2A). However, treatment with trastuzumab, a selective HER2 inhibitor, did not affect the paclitaxel resistance of SKOV3-TR and cell viability (Figure 3), suggesting that paclitaxel resistance was not affected due to the effect of dacomitinib on phosphorylation of HER2. Previous studies have reported that the expression of HER3 and HER4 was not observed in SKOV3 cells, the parent cells of SKOV3-TR [96,97]. Although we did not analyze the activities of HER3 and HER4 signaling mechanisms by dacomitinib in SKOV3-TR, we expected that the effect of treatment with dacomitinib on SKOV3-TR cells would be mainly due to the inhibition of EGFR and its downstream signaling mechanisms.

We investigated the effect of dacomitinib on P-gp as a mechanism to suppress paclitaxel resistance and induce apoptotic cell death by paclitaxel treatment in SKOV3-TR cells. ATP-dependent drug efflux transporter P-gp prevents cellular accumulation of their substrates, including paclitaxel and cisplatin, and causes multidrug resistance [98]. It has been reported that an EGFR inhibitor, gefitinib, inhibits the function of P-gp in multidrug-resistant lung cancer and breast cancer [99]. Xu L et al. reported that dacomitinib can suppress cisplatin resistance in cisplatin-resistant ovarian cancer cells by inhibiting the expression of P-gp [38]. Therefore, we suspected that dacomitinib might affect the expression or function of P-gp by combination treatment of dacomitinib and paclitaxel in SKOV3-TR cells. However, our data indicated no effect on P-gp by dacomitinib (Figure 4). We did not rule out the possibility that these results may be due to differences in cell types.

It has been known that paclitaxel-induced cell death requires intrinsic apoptotic pathways via Bcl-2 family proteins, but the mechanisms are not clearly defined [100]. Our data showed that the cellular level of Mcl-1, one of the prosurvival/antiapoptotic Bcl-2 family proteins, was clearly decreased in combination treatment with dacomitinib and paclitaxel in SKOV3-TR cells (Figure 5A). As several studies have reported that the regulation of cellular Mcl-1 levels is involved in the EGFR signaling mechanism and its effects on cell survival and chemoresistance [101,102,103], we expected that the expression level of Mcl-1 would be related to EGFR signaling in SKOV3-TR cells. First, it has been reported that the intracellular inhibition of Mcl-1 can overcome EGFR resistance. H Zang et al. reported that Mcl-1 degradation resensitized to osimertinib, a selective EGFR inhibitor, in EGFR-mutant lung cancer cells [104]. Recently, it has been reported that the inhibition of Mcl-1 and activation of Bax overcome acquired resistance to third-generation EGFR inhibitor osimertinib [105]. Second, there are reports that some EGFR tyrosine kinase inhibitors can inhibit the expression of Mcl-1 and induce apoptotic cell death. Yu X et al. reported that formononetin inhibits cell growth by suppressing EGFR signaling and reducing Mcl-1 in NSCLC [106]. Gao F et al. indicated that deguelin suppresses NSCLC cells by EGFR signaling via destabilization of Mcl-1 [107]. Our data showed dacomitinib treatment alone at sublethal concentrations did not affect the cellular level of Mcl-1 and also showed a decrease in Mcl-1 when combined with paclitaxel (Figure 5A), indicating there is a possibility that dacomitinib may indirectly reduce the cellular Mcl-1 during the apoptosis process. We considered that the decrease in cellular Mcl-1 plays an important role in apoptotic cell death by inhibiting the EGFR signaling mechanism. We are planning additional studies using transcriptome and proteome analysis to analyze the effect not only of the EGFR downstream genes but also various signaling pathways at the whole genome-wide level. Accumulating evidence indicates that ROS can be increased in cancer cells due to intracellular changes, such as metabolic reprogramming and mutations in ROS regulators, and environmental changes, including hypoxic conditions [39]. Increased levels of ROS are associated with tumor growth and response to therapy [108]. Paradoxically, ROS promotes tumorigenesis in cancer cells at low concentrations but also causes cell death at high concentrations [40]. Several anticancer drugs, including cisplatin and buthionine sulfoximine, act directly on ROS generation or inhibit the anticancer process, causing excessive ROS accumulation and directly leading to cell death [44,109]. Recently, it has been reported that the inhibition of EGFR signaling induces excessive intracellular ROS in cancer cells, leading to apoptosis [110]. Yan S et al. reported that FGFC1, a natural alkaloid, suppressed the growth of EGFR-mutant NSCLC cells by accumulating intracellular ROS via inhibition of the EGFR/PI3K/AKT/mTOR pathway [111]. Ge X et al. reported that almonertinib, an EGFR inhibitor, induces apoptosis and autophagy mediated by ROS in NSCLC cells [112]. Also, previous reports have indicated that paclitaxel has cell cytotoxic activity by inducing excessive intracellular ROS [48,113,114]. Li M et al. also reported that paclitaxel promotes apoptosis through the induction of ROS in osteosarcoma cells [114]. Ren X et al. reported that paclitaxel induces apoptosis through the regulation of the AKT/MAPK signaling pathway and ROS in a canine tumor [113]. These previous reports that excessive ROS can be induced during paclitaxel-induced apoptosis as well as inhibition of EGFR signaling led us to investigate whether intracellular ROS was excessively induced in SKOV3-TR cells cotreated with dacomitinib and paclitaxel. Our results showed that only the combination treatment of dacomitinib and paclitaxel induced excessive ROS in SKOV3-TR cells (Figure 5A). Moreover, NAC treatment alleviated paclitaxel-induced cytotoxicity in dacomitinib-treated SKOV3-TR cells, and these results indicated that paclitaxel induced a dose-dependent excessive ROS increase (Figure 5B). Our data showed that ROS was increased by paclitaxel in cells treated with sublethal concentrations (up to 1 μM) of dacomitinib, which did not induce ROS, but the detailed signaling mechanisms that induce ROS in SKOV3-TR cells are still unclear. Figure 1A indicates that treatment with dacomitinib above 1 μM induces cell cytotoxicity in SKOV3-TR cells. In additional experiments, we observed that the cell cytotoxicity induced at concentrations above 1 μM of dacomitinib was attenuated by NAC treatment, even in the absence of paclitaxel (Figure S2), indicating that dacomitinib can affect the signaling mechanisms that regulate ROS in SKOV3-TR cells. We are analyzing to determine whether the inhibition of EGFR and its downstream signaling by dacomitinib can induce ROS directly or indirectly and the synergistic mechanism of dacomitinib and paclitaxel in SKOV3-TR cells through further studies. In several studies, excessive ROS levels are appropriately suppressed by regulating signaling factors such as forkhead homeobox type O (FOXO), erythroid-2-related factor 2 (NRF2), and nuclear factor kappa-light-chain-enhancer of activated B cells (NF-κB) [115,116,117,118,119,120]. It has also been reported that these factors affect chemoresistance in various cancers [121,122,123,124].

In conclusion, these data indicated that dacomitinib downregulates the expression level of Mcl-1 and phosphorylation of Bad in SKOV3-TR cells through inhibition of the EGFR signaling pathway and resensitizes to paclitaxel, resulting in ROS-induced apoptosis. Our study suggests that dacomitinib presents a therapeutic option to overcome chemoresistance to paclitaxel, but it also provides information regarding druggable targets for chemoresistant ovarian cancer.

## 4. Materials and Methods

### 4.1. Cell Culture and Reagents

The human ovarian cancer cell line SKOV3 and paclitaxel-resistant ovarian cancer lines SKOV3-TR and HeyA8-MDR were used. SKOV3, SKOV3-TR, and HeyA8-MDR cells were generously supplied by Professor A.K. Sood (University of Texas MD Anderson Cancer Center, Houston, TX, USA). These cells were cultured in Roswell Park Memorial Institute’s (RPMI) 1640 medium (Biowest, Nuaillé, France) supplemented with 10% (*v*/*v*) fetal bovine serum (Corning, NY, USA) and 1% penicillin/streptomycin (Thermo Fisher Scientific, Waltham, MA, USA) at 5% CO_2_ and 37 °C. Paclitaxel-resistant cells were cultured with 50 nM of paclitaxel (Cayman Chemical Company, Ann Arbor, MI, USA). N-acetylcysteine (NAC) (Sigma-Aldrich, St. Louis, MO, USA) was used to free radical scavengers. Dacomitinib was purchased from MedChem Express (Monmouth Junction, NJ, USA). Dulbecco’s Phosphate-Buffered Saline (DPBS, WelGENE, Seoul, Republic of Korea) and dimethyl sulfoxide (DMSO, Sigma-Aldrich, Darmstadt, Germany) were used in this experiment.

### 4.2. Lactate Dehydrogenase (LDH) Assay

The EZ-LDH kit (DoGen, Seoul, Republic of Korea) was used to measure the cytotoxicity of dacomitinib to SKOV3-TR cells. Cells were seeded into individual wells of 96-well cell culture plates at a density of 4.0 × 10^3^ cells per well, followed by incubation at 5% CO_2_ and 37 °C for 24 h. Dacomitinib was treated by concentration (0.1, 0.5, 1, 5, and 10 μM) and incubated at 5% CO_2_ and 37 °C for 48 h. This assay was performed according to the manufacturer’s instructions. The absorbance was measured at 450 nm using Synergy™ HTX Multi-Mode Microplate Reader (Bioteck, Winooski, VT, USA).

### 4.3. Water-Soluble Tetrazolium Salt-1 (WST-1) Assay

Cell viability was measured by WST-1 assay. SKOV3-TR and HeyA8-MDR cells were seeded into individual wells of 96-well cell culture plates at a density of 6.0 × 10^3^ cells per well, followed by incubation at 5% CO_2_ and 37 °C for 24 h. The chemicals were treated for a duration of 48 h, and the concentrations of each chemical were administered as detailed below: In Figure 1B and Figure 7A, dacomitinib (0, 0.1, 0.5, 1 μM) and paclitaxel (0–500 nM) were applied either individually or in combination. In Figure 3B, trastuzumab (0, 10, 50, 100 μg/mL) and paclitaxel (0–500 nM) were administered either individually or in combination. In Figure 6B, NAC (5 mM) and paclitaxel (0–500 nM) were treated either individually or in combination. In Figure 6C, dacomitinib (1 μM), paclitaxel (0–500 nM), and NAC (5 mM) were applied either individually or in combination. In Figure S2A, dacomitinib (0–10 μM) and NAC (5 mM) were treated either individually or in combination. In Figure S2B, dacomitinib (0–10 μM), paclitaxel (200 nM), and NAC (5 mM) were administered, respectively. After removing the supernatant, cells were washed with DPBS. Ten percent EZ-Cytox (DoGen, Seoul, Republic of Korea) solution was divided into each well and incubated at 5% CO_2_ and 37 °C for 30 min. The absorbance was measured at 450 nm using Synergy™ HTX Multi-Mode Microplate Reader. NAC was diluted in DPBS and treated at 5 mM of concentration in cells for 48 h.

### 4.4. Crystal Violet Assay

Crystal violet assay was performed to indirectly evaluate cell viability by visualizing living cells. The paclitaxel-resistant ovarian cancer cells were seeded into individual wells of 96-well cell culture plates at a density of 4.0 × 10^4^ cells per well, followed by incubation at 5% CO_2_ and 37 °C for 24 h. The chemicals were treated for a duration of 48 h, and the concentrations of each chemical were administered as detailed below: In Figure 1C and Figure 7B, dacomitinib (0–10 μM) alone or dacomitinib (0.5 and 1 μM) and paclitaxel (0–400 nM) were treated either individually or in combination. In Figure 6D, paclitaxel (0–400 nM) was treated alone or in combination with NAC (5 mM) and dacomitinib (1 μM). In Figure S2C, dacomitinib (0–10 μM) was treated alone or in combination with NAC (5 mM) and paclitaxel (200 nM). After removing the supernatant, the cells were washed twice with DPBS. A 0.2% crystal violet (Biopure, Seoul, Republic of Korea) solution was added to each well, and cells were stained for 1 h at room temperature.

### 4.5. Immunoblotting and Antibodies

SKOV3, SKOV3-TR, and HeyA8-MDR cells were seeded in 100 mm culture plates at a density of 7.0 × 10^5^ cells, followed by incubation at 5% CO_2_ and 37 °C for 24 h. Cells were treated alone or in parallel with dacomitinib (1 μM) and paclitaxel (200 nM) or combined with dacomitinib (0.1, 0.5, and 1 μM) and paclitaxel (10, 100, and 200 nM) and then incubated at 37 °C in 5% CO_2_ conditions for 48 h. Cells were lysed using a RIPA lysis buffer (25 mM Tris-Cl, 5 mM EDTA, 1% NP40, 0.025% SDS, 150 mM NaCl, and 1% sodium deoxycholate) containing phosphatase inhibitor PhosSTOP™ (Roche, Basel, Switzerland). Protein concentrations were quantified using Pierce™ BCA Protein Assay Kits (Thermo Fisher Scientific, MA, USA). Extracts were separated by sodium dodecyl sulfate polyacrylamide gel electrophoresis (SDS-PAGE) and transferred to a 0.2 µm nitrocellulose membrane (Cytiva, Amersham, UK). The nitrocellulose membrane was blocked in 2% skim milk (Biopure, Cambridge, MA, USA) for 90 min and subsequently incubated with primary antibodies at 4 °C overnight. After washing 3 times for 5 min with 1X Tris-buffered saline buffer containing Tween 20 (1X TBST buffer), the secondary antibody was attached at room temperature for 2 h. After washing 3 times for 10 min with 1X TBST buffer, the secondary antibodies were detected by Clarity Western ECL Substrate (Biorad, Hercules, CA, USA). Actin was used as the loading control. The following antibodies were used: Akt (#9272), Phospho-Akt (Ser473) (#9271), p44/42 MAPK (ERK1/2) (#9102), Phospho-p44/42 MAPK (ERK1/2) (Thr202/Tyr204) (#9101), p38 MAPK (#9212), phosphor-p38 MAPK (Thy180/Tyr182) (#9211), Stat3 (124H6) Mouse mAb (#9139), phosphor-Stat3 (Yyr705) (D3A7) XP^®^ Rabbit mAb (#9145), Phospho-Stat3 (Ser727) (D8C2Z) Rabbit mAb (#92994), EGF Receptor (#2232), Phospho-EGF Receptor (Yyr1068) (#2234), HER2/ErbB2 (29D8) Rabbit mAb (#2165), Phospho-HER2/ErbB2 (Tyr1248) (#2247), Bcl-xL (54H6) Rabbit mAbB (#2764), Mcl-1 (D35A5) Rabbit mAb (#5453), Bax (D2E11) Rabbit mAb (#5023), Phospho-Bad (Ser155) (#9297), Caspase-3 (8G10) Rabbit mAb (#9665), Cleaved Caspase-3 (Asp175) (5A1E) Rabbit mAb (#9664), Cleaved PARP (Asp214) (D64E10) XP^®^ Rabbit mAb (#5625), and MDR1/ABCB1 (D3H1Q) Rabbit mAb (#12693S) were purchased from Cell Signaling Technology (Beverly, MA, USA). Bcl-2 Rabbit mAb (A19693) was obtained from ABclonal (Woburn, MA, USA). Bad (C-7) (sc-8044) and β-actin (C4) (sc-47778) were obtained from Santa Cruz Biotechnology (Dallas, TX, USA).

### 4.6. Flow Cytometry

Flow cytometry analysis was conducted to evaluate the effect of dacomitinib and paclitaxel on cell cycle arrest or apoptosis in SKOV3-TR cells. The cells were seeded in 100 mm culture plates at a density of 7.0 × 10^5^ cells, followed by incubation at 5% CO_2_ and 37 °C for 24 h. Dacomitinib (1 µM) and paclitaxel (200 nM) were treated individually or in combination, followed by incubation at 5% CO_2_ and 37 °C for 48 h. Then, 1 mL of ice-cold 70% ethanol was added to the cell pellet, one drop at a time, and made into a single-cell state. The cells were incubated at −20 °C for 1 h. The cells were centrifuged at 600× *g* for 2 min. After removing the supernatant, the cell pellet was washed with 1 mL of DPBS. Subsequently, the cells were treated with a 100 mg/mL propidium iodide solution (Sigma-Aldrich, Darmstadt, Germany) and incubated at room temperature for 30 min. After the sample was transferred to the fluorescence-activated cell sorting (FACS) sample tube, flow cytometry was analyzed immediately using FACS analysis (BD Bioscience, Mountain View, CA, USA).

### 4.7. Reverse Transcription Polymerase Chain Reaction (RT-PCR)

SKOV3-TR cells were seeded in 100 mm culture plates at a density of 7.0 × 10^5^ cells, followed by incubation at 5% CO_2_ and 37 °C for 24 h. Then, the cells were treated with dacomitinib (1 µM) and paclitaxel (200 nM) individually or in combination, followed by incubation at 5% CO_2_ and 37 °C for 48 h. Total RNAs were isolated from the cells using the Ribo-EX (GeneAll Biotechnology Co., Ltd., Seoul, Republic of Korea). Complementary DNAs were generated from total RNA using M-MLV-Reverse Transcriptase (Invitrogen, Waltham, MA, USA) with 0.1 M DTT and 5X First Strand Buffer. RT-PCR was performed using Taq DNA Polymerase, 2.5 mM dNTP mixture, 10X reaction buffer with MgCl_2_ (Bioneer, Daejeon, Republic of Korea), and DMSO. RT-PCR conditions were 30 s at 94 °C for denaturation, 30 s at 54–64 °C for annealing, and 60 s at 72 °C for extension. The primers used in this experiment are listed in Table 1.

### 4.8. Fluo-3/Acetoxymethyl (AM) Assay

SKOV3-TR or SKOV3 cells were seeded in 100 mm culture plates at a density of 7.0 × 10^5^ cells, followed by incubation at 5% CO_2_ and 37 °C for 24 h. Dacomitinib (1 µM) and paclitaxel (200 nM) were treated individually or in combination, followed by incubation at 5% CO_2_ and 37 °C for 48 h. Four micro-mole fluo-3/AM (Sigma-Aldrich, Darmstadt, Germany) solution was added and incubated at 37 °C for 1 h. The cells were observed through an inverted fluorescence microscope.

### 4.9. 2′-7′-Dichlorodihydrofluorescein Diacetate (DCFH-DA) Assay

DCFH-DA (Sigma-Aldrich, Darmstadt, Germany) was used to detect the generation of reactive oxygen species (ROS). SKOV3-TR cells were seeded in 24-well cell culture plates at a density of 2 × 10^4^ cells and incubated at 37 °C for 24 h. Dacomitinib (1 µM), paclitaxel (200 nM), and NAC (5 mM) were treated individually or in combination, followed by incubation at 5% CO_2_ and 37 °C for 48 h. The cells were washed with DPBS. A total of 10 µM of DCFH-DA solution was divided into each well for 30 min at 37 °C. The absorbance was measured using a fluorescence microplate reader at excitation wavelengths of 485 nm and emission wavelengths of 520 nm.

### 4.10. Statistical Analysis

Statistical analyses were conducted in triplicate, and the data are expressed as mean ± standard deviation (SD). Normally distributed data were evaluated using a one-way analysis of variance (ANOVA) followed by Tukey’s test, as specified in the figure legends. Values of *p* < 0.05 were considered statistically significant.

## Figures and Tables

**Table 1:** List of sequences used for RT-PCR.

Targets	Sequence of Primer
*EGFR*	F: 5′-CGCAAGTGTAAGAAGTGCGAA-3′
	R: 5′-CGTAGCATTTATGGAGAGTGAGTCT-3′
*HER2*	F: 5′-TAAGGACCCTCCCTTCTGCG-3′
	R: 5′-AAAGACCACCCCCAAGACCA-3′
*AKT*	F: 5′-ATGAGCGACGTGGCTATTGTG-3′
	R: 5′-GAGGCCGTCAGCCACAGTCTG-3′
*STAT3*	F: 5′-GATCCAGTCCGTGGAACCAT-3′
	R: 5′-TGGTCTTCAGGTATGGGGCA-3′
*ERK*	F: 5′-CCTAAGGAAAAGCTCAAAGA-3′
	R: 5′-AAAGTGGATAAGCCAAGAC-3′
*p38*	F: 5′-GATCAGTTGAAGCTCATTTTAA-3′
	R: 5′-CACTTGAATAATATTTGGAGAGT-3′
*BCL-2*	F: 5′-ATCGCCCTGTGGATGACTGAGT-3′
	R: 5′-GCCAGGAGAAATCAAACAGAGGC-3′
*BCL-XL*	F: 5′-GTAAACTGGGGTCGCATTGT-3′
	R: 5′-TGGATCCAAGGCTCTAGGTG-3′
*MCL-1*	F: 5′-CTCCCCACCAAGAGTCCACA-3′
	R: 5′-CCCAAACCACTTGGGGTGTC-3′
*BAX*	F: 5′-CCAGCTGCCTTGGACTGT-3′
	R: 5′-ACCCCCTCAAGACCACTCTT-3′
*MDR1*	F: 5′-GCGAGGTCGGAATGGATCTT-3′
	R: 5′-AGGGTTAGCTTCCAACCACG-3′
*GAPDH*	F: 5′-GTCTCCTCTGACTTCACAGCG-3′
	R: 5′-ACCACCCTGTTGCTGTAGCCAA-3′

## Data Availability

Data are contained within the article and Supplementary Materials.

## Supplementary Materials

The following supporting information can be downloaded at: https://www.mdpi.com/article/10.3390/molecules29010274/s1, Figure S1: The expression of P-gp between paclitaxel-sensitive SKOV3 and -resistant SKOV3-TR cells, Figure S2: N-acetylcysteine (NAC) restored dacomitinib-induced apoptosis in SKOV3-TR cells.
