# Supplementary material for: Pan-EGFR Inhibitor Dacomitinib Resensitizes Paclitaxel and Induces Apoptosis via Elevating Intracellular ROS Levels in Ovarian Cancer SKOV3-TR Cells
Source: Molecules. 2024 Jan 4;29(1):274. doi: 10.3390/molecules29010274 (PMC10780346; doi:10.3390/molecules29010274)
Supplement: Supplementary file 1 [file molecules-29-00274-s001.zip › molecules-2712913-supplementary.pdf]

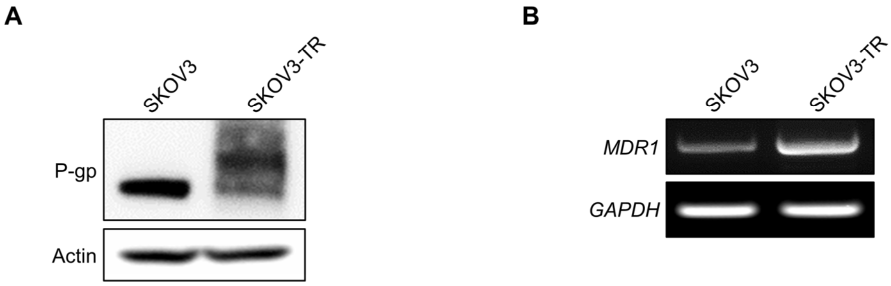

Figure S1. The expression of P-gp between paclitaxel-sensitive SKOV3 and -resistant SKOV3-TR cells. (A) SKOV3 or SKOV3-TR cells were seeded at 7.0 x 10^5 each on 100-mm culture plates and incubated for 48 h . The expression levels of P-gp were analyzed through immunoblotting. (B) Transcription levels of *MDR1* were analyzed by RT-PCR in each cell lines. *GAPDH* was used as a loading control for the mRNAs of oth er genes. Actin was used as a loading control for other proteins. DAC, Dacomitinib; PTX, Paclitaxel.

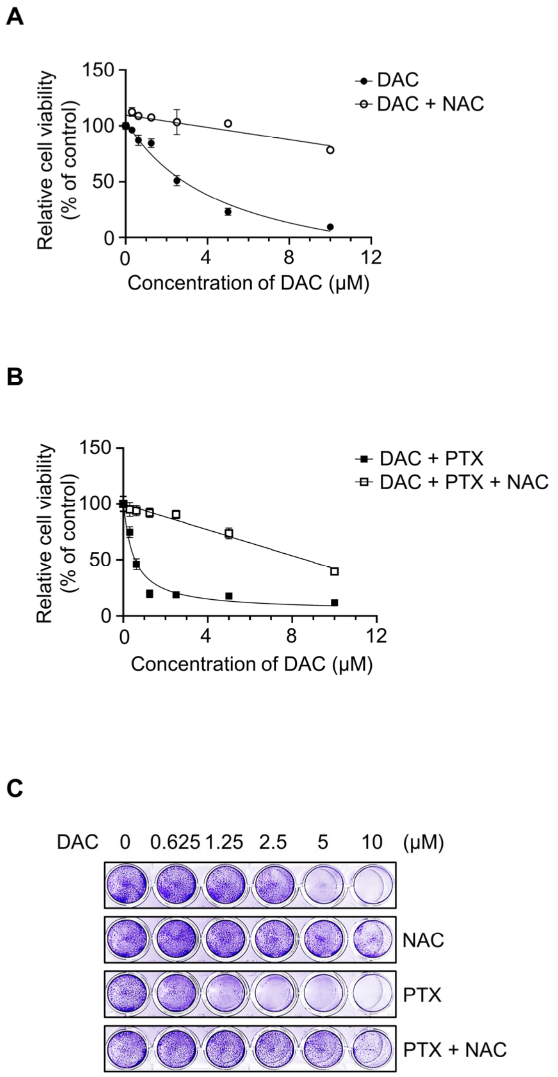

Figure S2. N-acetylcysteine (NAC) restored dacomitinib-induced apoptosis in SKOV3-TR cells. (A) SKOV3-TR cells were treated with serially diluted dacomitinib concentrations (0-10 μM) for 48 h, or serially diluted dacomitinib concentrations (0-10 μM) and N-acetylcysteine (5 mM) for 48 h, and cell viabilities were assessed using the WST-1 assay. (B) SKOV3-TR cells were treated with serially diluted dacomitinib concentrations (0-10 μM) and paclitaxel (200 nM), or serially diluted dacomitinib concentrations (0-10 μM) and paclitaxel (200 nM) and N-acetylcysteine (5 mM) for 48 h, and then cell viabilities were assessed using the WST-1 assay. (C) SKOV3-TR cells were cotreated with serially diluted dacomitinib concentrations (0-10 μM), serially diluted dacomitinib concentrations (0-10 μM) and paclitaxel (200 nM), serially diluted dacomitinib concentrations (0-10 μM) and paclitaxel (200 nM) and N-acetylcysteine (5 mM) for 48 h, and then stained with 0.2% crystal violet solution for 1 h at room temperature. DAC, Dacomitinib; PTX, Paclitaxel; NAC, N-acetylcysteine.
